# Surveillance and monitoring program of child neurodevelopment in population born during social confinement due to covid contigence: monteria-colombia experience

**DOI:** 10.1192/j.eurpsy.2024.681

**Published:** 2024-08-27

**Authors:** C. Otalvaro, A. Romero, M. C. Florian

**Affiliations:** ^1^Cordoba, Universidad de Córdoba; ^2^Cordoba, Georges Noble School, Monteria, Colombia

## Abstract

**Introduction:**

The American Academy of Pediatrics reports an incidence of 1 in every 54 children (Council on Children with Disabilities, 2021). The unique circumstances surrounding children born in 2020, who have experienced the COVID-19 pandemic since birth, present a distinct set of challenges for their neurodevelopmental well-being. The pandemic has led to reduced opportunities for learning and social interaction, masking mandates, decreased social support for research, and the potential misattribution of Autism Spectrum Disorders ASD symptoms to the effects of social isolation.

**Objectives:**

This study aims to develop such a program for children born during the COVID-19 pandemic (2020-2022).

**Methods:**

All children born in March 2020 were included in the study. The initial assessment involved administering the ASQ-3 to evaluate their development across the specified domains. Diagnostic Evaluation: Among the population, 6% (4 children) displayed concerning signs on the ASQ-3, warranting further diagnostic evaluation by specialized health professionals for possible ASD.

**Results:**

Early Intervention and School Monitoring: Of the remaining 72% (46 children), who did not require diagnostic evaluation, intervention guidelines were provided, both within the school environment and at home. These children were reevaluated after a three-month period. Follow-up in the School Environment: Those children who underwent reevaluation were categorized into three groups: Nine children fell into the “gray” category on the ASQ-3 and were subsequently referred for diagnostic evaluation. Thirty-seven children progressed to the “white” category on the ASQ-3 after receiving intervention guidelines in both school and home settings. The findings of this research underscore the potential impact of the COVID-19 pandemic on the neurodevelopment of children born in 2020. 6% of the evaluated population were referred for diagnostic evaluation due to signs of ASD, suggesting a potential association between the pandemic and an increased risk of ASD within this cohort.72% of children who received intervention guidelines demonstrated significant improvements in their neurodevelopment, highlighting the critical role of early intervention and school-based monitoring

**Conclusions:**

Implementing support strategies within educational settings was linked to positive developments in neurodevelopmental outcomes. Consequently, school-based neurodevelopmental monitoring, complemented by cohesive curricular guidelines, emerges as a beneficial approach for enhancing child development outcomes. The ASQ-3, as a structured instrument, proves invaluable in facilitating neurodevelopmental surveillance within educational settings, particularly in contexts with high demand and limited access to specialized care.

**Disclosure of Interest:**

None Declared

